# 

**Published:** 1890-03-29

**Authors:** 


PRICE ONE PENNY.

No. 183, Vol. VII.—March 29th, 1890.

[Registered at the General Post Office as a Newspaper for transmission in the United Kingdom and Abroad.

WITH EXTRA NURSING SUPPLEMENT
Per Annum, 6s. 6d.; post free.
Monthly Parts, 6d.; post free 8d.
Ditto per Annum 8s. post free.

### THE HOSPITAL

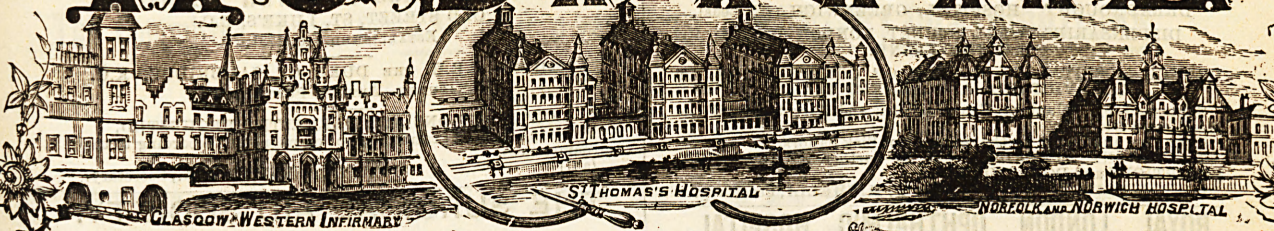

A WEEKLY JOURNAL OF

### Science, Medicine, Nursing and Philanthropy.

SATURDAY, MARCH 29th, 1890.

**The Doctor's Domestic Intellect** - 399
**Words of Consolation.**—Good Friday - 400
**The Doctor's Pulpit** —Out of Physic: Into—What? - 401
**Penitentiary Patients** - 402
**Everybody's Page** - 403
**Notes and News** - 404
**Annotations.**—M. Pasteur at Play—Specific Diseases: Infection - 406
**The Doctor in History** - 411
**Passing Topics.**—The Squandering of Posthumous Gifts to Charity - 412
**Scraps and Gleanings** - 412
**The Practitioner's Mirror and Retrospect:**
MEDICINE—Balsam of Peru in Phthisis—Treatment of Dengue—Noises in the Ears—Oil of Eucalyptus in Scarlatina and other Infectious Diseases—Dieulafoy on the Treatment of Asthma—Treatment of Goitre by Electricity—Antiseptic after Treatment of Vaccination - 407
THERAPEUTICS—Dangers of Sulphonal—A True Stomachic—Excipient for External Application - 408
THE PRACTITIONER'S BOOKSHELF.—History and Pathology of Vaccination - 409
NEW DRUGS, APPLIANCES, AND THINGS MEDICAL.—Robinson's Patent Groats and Patent Barley - 410

EXTRA SUPPLEMENT—THE NURSING MIRROR.

**En Passant.**—Bristol District Nurses—The Brassey Holiday Home—Short Items—Thousands of Pounds for the National Pension Fund—Frome Nurses' Home—The First Thousand—A Good Idea—American Notes - cv
**Notes from Australia** - cvi
**Nursing Lectures.** By PERCY LEWIS, M.D. VI.—The Abdomen - cvi
**Everybody's Opinion.**—Meum and Tuum - cvii
**Death in Our Ranks** - cviii
**Examination Questions** - cviii
**Appointments** - cviii
**Notes and Queries** - cviii
**Vacancies** - cviii
**Amusements and Relaxation** - cviii

